# Psychometric properties of the German version of Observer OPTION^5^

**DOI:** 10.1186/s12913-018-2891-6

**Published:** 2018-01-31

**Authors:** Mara Kölker, Janine Topp, Glyn Elwyn, Martin Härter, Isabelle Scholl

**Affiliations:** 1 Department of Medical Psychology, University Medical Center Hamburg-Eppendorf, Martinistr. 52, W26, 20246 Hamburg, Germany; 2 The Dartmouth Institute for Health Policy and Clinical Practice, Dartmouth College, Williamson Translational Research Building Level 5, 1 Medical Center Drive, Lebanon, NH 03756 USA

**Keywords:** Decision-making, Shared decision-making, Communication, Psychometrics, Measurement, Patient involvement

## Abstract

**Background:**

In order to conduct studies on shared decision-making (SDM) and to implement SDM in routine practice, psychometrically tested measures are needed. The development of the short 5-item version of the OPTION scale (Observer OPTION^5^) allows to assess SDM from an observer perspective. Observer OPTION^5^ is so far only available in English and Dutch. The aim of this study was to translate the Observer OPTION^5^ rating scale into German and to test its psychometric properties.

**Methods:**

The German Observer OPTION^5^ was tested in a secondary data analysis of audio-recordings of patient-physician-consultations (*N* = 79) in German primary care practices. Demographic data were analysed using descriptive statistics. To assess inter- and intra-rater reliability, intraclass correlation coefficients (ICCs) were calculated. For assessing concurrent validity, a correlation (Spearman’s Rho) of the sum score of Observer OPTION^5^ and Observer OPTION^12^ was calculated.

**Results:**

The consultations dealt with decisions regarding type 2 diabetes (*N* = 31), chronic back pain (*N* = 23), depression (*N* = 20), and other diseases (*N* = 5). Analysis of inter-rater reliability yielded an ICC of 0.82 for the sum score; across the five single items ICCs ranged between 0.45 and 0.77. For the intra-rater reliability an ICC of 0.83 was observed for the total score; across the five single items ICCs ranged between 0.45 and 0.86. The Observer OPTION^5^ had a mean total score of 11.84 (SD = 11.92) and the Observer OPTION^12^ had a mean total score of 10.3 (SD = 7.9), both on a potential range of 0 to 100. The correlation between the total scores of Observer OPTION^5^ and Observer OPTION^12^ was *r* = 0.47 (*p* = 0.01).

**Conclusions:**

The results regarding inter- and intra-rater reliability were excellent on the total score level. Observer OPTION^5^ showed moderate concurrent validity using Observer OPTON^12^. The results are generally comparable to the results of the original English version of Observer OPTION^5^. The German version of Observer OPTION^5^ can be used in research and evaluation of clinical practice. Nevertheless, further testing is adviced.

## Background

Over the last years, there has been a shift in physician-patient communication away from the paternalistic model of decision-making towards shared processes between physicians and patients [1, 2]. In the paternalistic model of decision-making, the physician is characterized as information keeper, who makes decisions for the patient in the intention to know what is best for the patient [1]. Shared decision-making (SDM) is defined as a collaborative process that allows a patient and his/her provider(s) to make health care decisions together based on shared clinical and psychosocial information and the best available evidence [3].In the course of this process, the provider(s) support(s) the patient to engage in deliberation about the different diagnostic or treatment options in order to come to a shared and informed decision in concordance with the patient’s informed preferences [3].

To evaluate whether SDM has been implemented in health care, the physicians’ communicative skills for sharing information and for involving patients in the decision-making process have to be assessed. Therefore, the development and psychometric testing of observer rating scales that evaluate whether SDM took place is essential to allow standardised evaluation of physician-patient communication.

Although preferences for participation in decision-making differ between patients with different diagnoses, most patients want to be involved if more than one treatment option exists [4–6]. SDM is positively associated with patient outcomes (e.g., knowledge, satisfaction, decisional conflict, trust) [7]. Despite patients’ preferences for SDM and its positive effects on patient outcomes, it is still not well implemented in routine practice [5, 8]. The discrepancy between patients seeking involvement and physicians obstructing this involvement can be analysed from a patient’s, a physician’s and an observer’s point of view [9, 10]. Observer rating can provide a general estimate of the involvement of both parties and permits an objective assessment of the SDM process in a consultation. Several observer rating scales exist in English, e.g. the Observer OPTION^12^,the Rochester Participatory Decision Making Scale, the Brief Decisison Support Analysis Tool, and the Decision Analysis System for Oncology [10].

So far the Observer OPTION^12^ (OPTION scale - observing patient involvement) is the only internationally widely used observer measure available in German language [10]. Until now the Observer OPTION^12^ is a frequently applied observer measure to assess SDM. The Observer OPTION^12^ can be used by trained observers to assess SDM during a consultation, in communication trainings or in research using recorded consultations. In the development of new scales Observer OPTION^12^ has been often used as a comparator scale to assess validity [11, 12]. Despite its wide use, psychometric testing of Observer OPTION^12^ revealed a great variation in reliability across different studies [13], and the necessity for improvements concerning specific items. Several items of Observer OPTION^12^ (mainly focusing on the degree of exploration of the patient’s preferences and checking the patient’s understanding) were rarely observed (i.e. mainly rated 0) or not specific for SDM [14]. Other items were revised or combined [14]. This led to the development of Observer OPTION^5^ as a shorter and revised version of Observer OPTION^12^ [14]. For the development of Observer OPTION^5^ published models were analysed to identify the core components of a conceptual framework of SDM. By using this framework, which includes data from an observational study of clinical practice in Canada and the existing experience of using Observer OPTION^12^, Observer OPTION^5^ was developed [14]. Observer OPTION^5^ focuses on the core aspects of SDM and is shorter with only 5 items. Thus, the scale may be less time-consuming and easier to implement in clinical settings [14]. Furthermore, the scale also assesses patient contribution to the decision-making process unlike the Observer OPTION^12^, which only assesses the physician’s contribution to the process. Both measures are described in more detail in the Methods section.

Psychometric testing of the English version of Observer OPTION^5^ showed adequate concurrent validity with Observer OPTION^12^ (*r* = 0.61), intra-rater reliability (*r* = 0.93) and inter-rater agreement (ICC = 0.67) [15]. A Dutch version reached comparable results with good inter-rater agreement (k = 0.68) and a positive correlation with Observer OPTION^12^ (*r* = 0.71) [16]. Based on results of these prior studies on the English and Dutch versions, we hypothesised that the German Observer OPTION^5^ version would reach comparable results [15, 16].

This study aimed to establish a German version of Observer OPTION^5^ and to test its psychometric characteristics.

## Methods

### Translation process

The original English version of the Observer OPTION^5^ was translated into German to reach cross-cultural equivalence between these versions [17]. In collaboration with the main developer (GE) of Observer OPTION^5^, we agreed on a translation process that consisted of a translation from the original English version to German by two independent bilingual translators (MK, WF (cp. Acknowledgements)), whose first language is German. In the next step, a third person (IS) suggested a third German version that combined the first two translations. Then the three translators reached consensus on one final version. This so-called ‘team translation approach’ does not include a backward translation [18], as a backward-translation does not necessarily reveal the major discrepancies of the original and targeted versions and provides no critical information regarding the underlying issue for the discrepancies [19, 20]. The final German version and the corresponding translated user manual were evaluated during the rater-training, which led to a subsequent revision of a few phrases in the manual. The German manual is available from the corresponding author upon request.

### Psychometric testing and study design

This study used audio-recordings of patient-physician consultations to assess SDM using the Observer OPTION^5^. These data were collected in a different study on the psychometric testing of the 9-item Shared Decision Making Questionnaire (SDM-Q-9), funded by the German Ministry of Education and Research. In 2010 patient-physician consultations in primary care (i.e. in private practice non-hospital settings) were audio-recorded as part of this study. Additionally, demographic data of patients and physicians were collected via self-report questionnaires. Furthermore, the physicans provided information about the patients’ diagnosis and reason for the consulation. Inclusion critera for patients were 1) a diagnosis of type 2 diabetes, chronic back pain or depression, 2) above 18 years of age, 3) German-speaking and 4) facing a treatment decision in consideration of one of the three diagnoses named above. Patients with cognitive impairment were excluded. Few physicians with problems in including patients were instructed to include patients with other chronic diseases (e.g. hypertension). Most recorded consultations dealt with one specific decision, since this was the instruction for participating physicians [11].

Within the primary study, the recordings were evaluated with Observer OPTION^12^; these existing ratings of the Observer OPTION^12^ were re-used in this study.. A total of 79 audio-recordings were now additionally rated in this secondary data-analysis with the German version of Observer OPTION^5^. In the primary study a sample size was aimed that would allow the detection of correlations above 0.5 with a power of 80% to provide a solid basis for the psychometric analyses. With an estimated dropout of 20% of physicians and missing data (estimated 12.5% of consultations), a final sample size of *N* = 63 was definited to be adequate in the first study [11].

### Rater training and rating process

The training of the two raters was undertaken by one of the authors (IS), who was trained for the rating of Observer OPTION^12^ and took part in a workshop on Observer OPTION^5^. Two reviewers (MKand JT), both familiar with the concept of SDM, were trained on how to use the Observer OPTION^5^ during a six-hour rater training. Five audio recordings and two video recordings were examined independently by all raters. The results were then discussed and consent was formed with help of the manual.

After the training the 79 records were evaluated separately by both raters in order to assess inter-rater reliability of the German version of Observer OPTION^5^. One of the raters (MK) rated them a second time within one month of the first rating to assess intra-rater reliability.

### Observer OPTION^12^ and Observer OPTION^5^ scale

The Observer OPTION^12^ scale measures the degree of perceived SDM in a consultation. It focuses on the physician’s SDM behaviour and can be used in various medical situations [13]. So far it has been translated into Chinese, Dutch, French, German, Italian, Spanish and Swedish [13]. The scale consists of 12 items measuring aspects of SDM, which can be rated on a 5-point Likert scale (from 0 = the behaviour is not observed to 4 = the behaviour is observed and executed to a high standard) [21, 22]. Psychometric testing showed good inter-rater reliability (ICC = 0.77) [21]. However, item independence requires further psychometric testing [13].

In Observer OPTION^5^ some of the Observer OPTION^12^ items were excluded or combined because the items were not specific enough for SDM or too idealistic to realise [14]. Furthermore, the Observer OPTION^5^ allows to rate a physician’s reaction if a patient actively brings up a part of the SDM process. This focus on the dyadic process was added to the Observer OPTION^5^ rating scale, as it was a shortcoming of the Observer OPTION^12^, where it was only possible to rate actions of physicians.The items of Observer OPTION^5^ regarding SDM are observer rated and include the following: 1) informing the patient that a decision has to be made, 2) assuring that the patient will be supported and deliberate about options, 3) giving information on the options and mentioning pros and cons, 4) eliciting the patient’s preferences, and 5) how to integrate the patient’s preferences in the decision. These five items can be rated on a 5-point Likert scale, which is shown in Table 1.

**Table 1:** Scoring Guide Observer OPTION^5^ [26]

Score	Description
0 = No effort	Zero effort observed.
1 = Minimal effort	Effort to communicate could be implied or interpreted.
2 = Moderate effort	Basic phrases or sentences used.
3 = Skilled effort	Substantive phrases or sentences used.
4 = Exemplary effort	Clear, accurate communication methods used.

### Data analysis

In this study 79 audio-recordings were included. Descriptive statistics were calculated for demographic data. To test intra- and inter-rater reliability intra-class correlation coefficients (ICC) were calculated. This included an overall score and an item-by-item testing. Regarding the overall score, the results were rescaled to a total score of 0 to 100. For the ICC calculation the two-way-mixed model was used. An absolute agreement and a mean ICC were assessed. The comparison of Observer OPTION^5^ to the previous Observer OPTION^12^ scale was examined by testing concurrent validity. Since no normal distribution was found, a Spearman’s correlation was calculated. Spearman’s Rho is examined by an averaged cumulative value. For all measures a formative measure model was used and all data were analysed with SPSS Statistics 23 (SPSS Inc., Chicago, IL). Results of ICC between 0.75–1.0 were classified as excellent, 0.6–0.74 as good, 0.4–0.59 as moderate and 0–0.39 as poor [23].

## Results

### Sample characteristics

The consultations dealt with decisions regarding type 2 diabetes in 31 consultations, chronic back pain in 23, depression in 20, hypertension in two and other diseases in three consultations. Two-thirds were female and one-third male patients and their mean age was 54.7 years. The majority of the patient sample had a low education level (52.6%) and were married (48%). Demographic and clinical characteristics are shown in Table 2.

**Table 2:** Characteristics of the participating patients (*N* = 79a)

	*N* = 79^a^	in %
Sex		
Female	51	65.4
Male	27	34.6
Age, years		
Mean (SD, range)	54.7 (14.82, 23–93)	
Education		
Low^b^	41	52.6
Medium^c^	27	34.6
High^d^	10	12.8
Occupation		
Employed	35	46.1
Retired	28	36.8
Unemployed	8	10.5
Homemaker	4	5.3
Student	1	1.3
Family status		
Married	36	48.0
Never Married	20	26.7
Divorced	13	17.3
Widowed	6	8.0
Health problem in rated consultation (physician reported)		
Type 2 diabetes	31	39.2
Chronic back pain	23	29.1
Depression	20	25.3
Hypertension	2	2.5
Other	3	3.8

^a^Sample size varies between 75 and 79 due to missing values

^b^Years of education completed ≤9

^c^Years of education completed 10–12

^d^Years of education completed ≥13

The physician sample included eleven (45.5%) general practitioners, eight (33.3%) specialists for internal medicine, three (12.5%) orthopaedics and two (8.2%) psychiatrists. Physicians’ mean age was 49.4 with a mean of eleven years of professional experience. In Table 3 additional information on participating physicians are displayed.

**Table 3:** Characteristics of the participating physicians

	*N* = 24	in %
Sex		
Female	11	54.2
Male	13	45.8
Age, years		
Mean (SD, range)	49.4 (8.62, 35–66)	
Profession		
General Practitioner	11	45.5
Internal Medicine	8	33.3
Orthopaedics	3	12.5
Psychiatrist	2	8.2
Years of professional experience		
Mean (SD, range)	11 (9.12, 1–33)	

### Psychometric results

The items were evaluated with a range on the total score of 0 to 47.5 after rescaling the scale to a total of 100. The average was 11.84 (SD 11.92) for the total score on the Observer OPTION^5^. Overall no item was rated with 4 = exemplary effort. The item frequencies are displayed seperately for each rater in Table 4 and Table 5. ICCs for the inter-rater reliability of single items ranged between 0.45 (item one) and 0.77 (item three). The overall inter-rater reliability observed an ICC of 0.82. For the intra-rater reliability the ICCs of single items lay between 0.45 (item two) and 0.86 (item one and three) and the total score reached an ICC of 0.83. Item two showed a deviating result with an ICC of 0.45. The results for inter- and intra-reliability and the mean item evaluations are displayed in Table 6.

**Table 4:** Item frequencies rater 1

Items	No effort^a^ (in %)	Minimal effort^a^ (in %)	Moderate effort^a^ (in %)	Skilled effort^a^ (in %)	Exemplary effort^a^ (in %)
Item 1: informing the patient that a decision has to be made	30(38)^b^/34(43)^c^	33(41.8)^b^/27(34.2)^c^	13(16.5)^b^/15(19)^c^	3(3.8)^b^/3(3.8)^c^	0(0)^b^/0(0)^c^
Item 2: assuring that the patient will be supported and deliberate about options	63(79.7)^b^/68(86.1)^c^	15(19)^b^/11(13.9)^c^	1(1.3)^b^/0(0)^c^	0(0)^b^/0(0)^c^	0(0)^b^/0(0)^c^
Item 3: giving information on the options and mentioning pros and cons	47(59.5)^b^/48(60.8)^c^	19(24.1)^b^/20(25.3)^c^	12(15.2)^b^/9(11.4)^c^	1(1.3)^b^/2(2.5)^c^	0(0)^b^/0(0)^c^
Item 4: eliciting the patient’s preferences	48(60.8)^b^/50(63.3)^c^	28(35.4)^b^/26(32.9)^c^	3(3.8)^b^/3(3.8)^c^	0(0)^b^/0(0)^c^	0(0)^b^/0(0)^c^
Item 5: integrating the patient’s preferences in the decision	53(67.1)^b^/51(64.6)^c^	26(32.5)^b^/25(31.6)^c^	0(0)^b^/3(3.8)^c^	0(0)^b^/0(0)^c^	0(0)^b^/0(0)^c^

^a^Sample size of *N* = 79 for each rating

^b^First rating

^c^Second rating

**Table 5:** Item frequencies rater 2

Items	No effort^a^ (in %)	Minimal effort^a^ (in %)	Moderate effort^a^ (in %)	Skilled effort^a^ (in %)	Exemplary effort^a^ (in %)
Item 1: informing the patient that a decision has to be made	61 (77.2)	14 (17.7)	4 (5.1)	0 (0)	0 (0)
Item 2: assuring that the patient will be supported and deliberate about options	70 (88.6)	8 (10.1)	1 (1.3)	0 (0)	0 (0)
Item 3: giving information on the options and mentioning pros and cons	42 (53.2)	18 (22.8)	12 (15.2)	7 (8.9)	0 (0)
Item 4: eliciting the patient’s preferences	40 (50.6)	27 (34.2)	9 (11.4)	3 (3.8)	0 (0)
Item 5: integrating the patient’s preferences in the decision	53 (67.1)	19 (24.1)	6 (7.6)	1 (1.3)	0 (0)

^a^Sample size of *N* = 79

**Table 6:** Inter- and Intra-rater reliability and mean item score

Items		Inter-rater reliability	Intra-rater reliability
	Mean (SD)	ICC (*N* = 79)	ICC (N = 79)
Item 1: informing the patient that a decision has to be made	0.57 (0.59)	0.45	0.86
Item 2: assuring that the patient will be supported and deliberate about options	0.17 (0.35)	0.61	0.45
Item 3: giving information on the options and mentioning pros and cons	0.69 (0.82)	0.77	0.86
Item 4: eliciting the patient’s preferences	0.56 (0.63)	0.71	0.69
Item 5: integrating the patient’s preferences in the decision	0.38 (0.53)	0.73	0.54
Total score^a^	11.84 (11.92)	0.82	0.83

^a^rescaled to a total score of 0 to 100

**Fig. 1:**
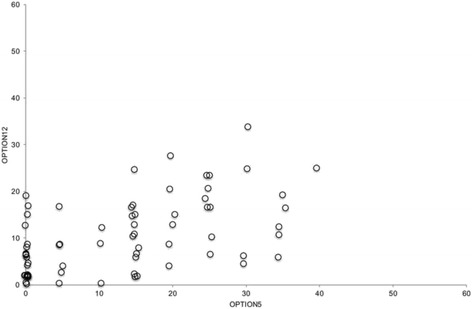
Scatterplot of Observer OPTION^5^ and Observer OPTION^12^ Total Scores

A significant correlation (*p* = 0.01) between the Observer OPTION^5^ total score and Observer OPTION^12^ total was observed (*r* = 0.47). This shows a positive correlation. A scatterplot of the sum scores of both scales are shown in Fig. 1.

## Discussion

In this study a German version of the Observer OPTION^5^ scale was developed and psychometrically tested. As part of a secondary data analysis, audio recordings of primary care consultations were evaluated independently by two raters with the German Observer OPTION^5^. Comparable results to the English and Dutch version were hypothesised [15, 16]. The testing of the German version of Observer OPTION^5^ showed excellent inter- and intra-rater reliability on the total score levels (0.82 and 0.83). On the item level, the inter-rater and intra-rater reliabilities were moderate to excellent (0.45–0.86). No item was rated higher than three (=skilled effort), leading to a left-skewed distribution, which is comparable to the first psychometric testing of Observer OPTION^5^ [15]. This result might be influenced by the physician sample, as none of the participating physicans had any particular training in SDM. A systematic review on studies using Observer OPTION^12^ found similarly low ratings in untrained healthcare providers [22].

The results regarding reliability are comparable to the first psychometric testing of the original English version of Observer OPTION^5^ (ICC = 0.67) [15] and the psychometric testing of the Dutch version (k = 0.68) [16]. These high inter-rater reliability results (ICC = 0.82) in this study compared to inter-rater agreement (ICC = 0.67) in the first Observer OPTION^5^ testing of the English version [15] may be due to differences in the determination of the relevant decision. In the study at hand, mostly one main decision was dealt with in the consultations. In other studies, vague or many decisions within one consultation may cause lower inter-rater agreement, because raters might not focus on the same issue. The assessment of concurrent validity of the German Observer OPTION^5^ scale compared to Observer OPTION^12^ showed a moderately positive correlation. While the concurrent validity using a correlation to Observer OPTION^12^ (*r* = 0.47) is a bit lower than in the two other studies (*r* = 0.61; *r* = 0.71) [15, 16], we still found a significant moderate positive correlation [24], which is in line with our hypothesis. The comparatively smaller correlation might be influenced by the low variance in the Observer OPTION^5^ scores, which is known to deteriorate measures of association (also referred to as the ‘restriction of range’ problem).

These psychometric results indicate that the German version of Observer OPTION^5^ is a reliable and valid rating scale. It is the shortest available observer rating scale for SDM. This scale can be used to assess SDM in physician-patient-communication and to evaluate physicians’ communication skills. Furthermore, as suggested by Barr and colleagues [15], the Observer OPTION^5^ could possibly be used in communication trainings for physicians as a feedback tool to improve physicians’ SDM skills. However, further research the measure’s potential use as training tool is necessary.

A main strength of this study was the widespread assessment of psychometric properties including inter-rater, intra-rater and concurrent validity of the newly adapted German Observer OPTION^5^. Since testing showed positive agreement between the German Observer OPTION^5^ scale and the previous Observer OPTION^12^ scale, the German Observer OPTION^5^ was shown to be feasible for use as an observer rating scale in German speaking countries.

A limitation of this study is that the evaluated data showed low variance. The items were mostly rated with no effort (0) or minimal effort (1). Nevertheless, this study reached good psychometric results for inter-rater agreement, intra-rater agreement and concurrent validity. Furthermore, the psychometric properties of the German version of Observer OPTION^5^ were tested in an primary care setting with encounters focussing mainly on three chronic conditions. Generalizability beyond this setting is limited. Whenever a measure is used in a different setting, a different patient group or a different country psychometric properties should be re-established [25]. Future studies should investigate other psychometric properties like responsiveness in order to establish a scale that can be used in intervention studies in the future. It would also be important to test Observer OPTION^5^ with a sample of physicians trained in SDM, in order to assess whether this leads to a higher variation of items distribution than in the present study.

## Conclusion

This study shows that the developed German version of Observer OPTION^5^ has good inter-rater and intra-rater agreement. Furthermore, the results indicate moderate concurrent validity of Observer OPTION^5^. These results support the body of evidence regarding the validity and reliability of the tool. It can be used to evaluate decision-making processes in clinical practice settings and in health services research. Nevertheless, further testing is advised, especially before using the measure in other settings or with other patient groups.

## Abbreviations

OPTION scale: Observing patient involvement scale
SDM: Shared decision-making
